# Evaluating the Presence and Contents of Phytochemicals in Honey Samples: Phenolic Compounds as Indicators to Identify Their Botanical Origin

**DOI:** 10.3390/foods10112616

**Published:** 2021-10-28

**Authors:** Lua Vazquez, Daniel Armada, Maria Celeiro, Thierry Dagnac, Maria Llompart

**Affiliations:** 1 CRETUS, Department of Analytical Chemistry, Nutrition and Food Science, Universidade de Santiago de Compostela, E-15782 Santiago de Compostela, Spain; lua.vazquez.ferreiro@usc.es (L.V.); daniel.armada.alvarez@usc.es (D.A.); maria.celeiro.montero@usc.es (M.C.); 2 Galician Agency for Food Quality-Agronomic and Agrarian Research Centre (AGACAL-CIAM), Unit of Food and Feed Safety and Organic Contaminants, Apartado 10, E-15080 A Coruña, Spain

**Keywords:** honey, polyphenols, phenolic profile, total phenolic content, antioxidant activity, liquid chromatography, tandem mass spectrometry, principal components analysis

## Abstract

Honey is a natural product well known for its beneficial properties. It contains phytochemicals, a wide class of nutraceuticals found in plants, including compounds with highly demonstrated antimicrobial and antioxidant capacities as phenolic compounds and flavonoids. The main goal of this work is the development of a miniaturized and environmentally friendly methodology to obtain the phenolic profile of Galician honeys (Northwest Spain) from different varieties such as honeydew, chestnut, eucalyptus, heather, blackberry and multi-floral. The total phenolic content (TPC) and antioxidant activity (AA) were also evaluated. As regards sample preparation, miniaturized vortex (VE) and ultrasound assisted extraction (UAE) employing aqueous-based solvents were performed. Individual quantification of 41 target phenolic compounds was carried out by liquid chromatography-tandem mass spectrometry (LC-MS/MS). Results revealed the presence of 25 phenolic compounds in the 91 analyzed samples, reaching concentrations up to 252 µg g^−1^. Statistical tools such as analysis of variance (ANOVA) and principal component analysis (PCA) were employed to obtain models that allowed classifying the different honeys according to their botanical origin. Obtained results, based on TPC, AA and ∑phenolic compounds showed that significant differences appeared depending on the honey variety, being several of the identified phenol compounds being responsible of the main differentiation.

## 1. Introduction

Honey is a natural food product well known not only for its nutritional value, but also for its antimicrobial, antiviral, antifungal, anticancer, and antidiabetic properties, as several in vitro and in vivo studies have demonstrated [1]. From a compositional point of view, honey is a highly concentrated solution of complex mixture of sugars: fructose (38%), glucose (31%), water (17%), maltose (7%), as well as trisaccharides, other higher carbohydrates, sucrose, minerals, vitamins, and enzymes. Its composition depends strongly on the plant species from which the nectar or the honeydew was collected, and other factors, such as postharvest treatments, geographical, environmental or climate conditions [2,3]. Honey is among the top ten foods with the highest adulteration rate in the European Union, that implies a detrimental to its quality and consumers safety [4]. To protect this valuable food, a Codex standard for honey was adopted by the Codex Alimentarius Commission in 1981, being further revised in 1987, 2001 and 2019, to regulate its production and storage, establishing parameters to guarantee its quality [5]. In 2001, the European Council, following the Codex recommendations, established the Directive 2001/110/EC [6], amended 2014/63/EU [7] that laid down the production and trading parameters of honey within the member states of the EU. However, several countries issue national provisions, decisions, and guidelines defining their own physicochemical, organoleptic and microscopic characteristics, enhancing the difficulties of the applicability of harmonized regulations [8].

The identification of honey botanical origin is a valuable information to assure honey quality. In this way, the analysis of its phenolic composition has been employed as a tool for its classification and authentication [1,9,10]. Phenolic compounds are secondary metabolites of plants generally involved in their defense against ultraviolet radiation or pathogens and have been recognized as the main responsible for the antioxidant activity of honey [11,12,13]. The most abundant phenol- types in honey are flavonoids, especially flavones and flavanols, as well as phenolic acids derived from benzoic and cinnamic acids [2,14].

Several analytical procedures have been reported to determine honey physicochemical properties including colour, viscosity, pH, moisture, free acidity, electrical conductivity, sugars, HMF (hydroxymethylfurfural) content, formol index and insoluble solids [15,16,17,18,19], but due to the high number of existing honey varieties, more specific techniques are needed. The use of chromatography coupled to mass detectors (MS) to obtain a deep chemical characterization of this product is a very valuable option. However, the major drawback for honey analysis is sample preparation since it is a very complex matrix. To establish the honey aromatic profile, the combination of solid-phase microextraction (SPME) with gas chromatography-mass spectrometry (GC-MS) has been the main employed technique [20,21,22]. On the other hand, for the determination of more polar analytes, including phenolic compounds, traditional sample preparation involves the use of solid-liquid or liquid-liquid (SLE, LLE) before LC-MS or HPLC-UV analysis. However, these techniques are long time consuming, requiring large amounts of organic solvents and further clean-up steps before analysis. Microwave-assisted extraction (MAE) and ultrasound assisted extraction (UAE) have been also proposed as extraction techniques to determine phenolic compounds in honey, but their use was not satisfactory in the presence of thermosensitive flavonoids such as quercetin, kaempferol or myricetin, that are almost degraded as consequence of radiation. On the other hand, both extraction techniques seemed to be suitable for the extraction of phenolic acids [20].

Therefore, the goal of this work is the development of a miniaturized analytical methodology to obtain the phenolic profile of Galician honeys (Northwest Spain) from different varieties and nectar sources. A green, fast and low-cost sample preparation strategy based on vortex extraction (VE) followed by ultrasound assisted extraction (UAE) employing aqueous- based solvents was assessed. Individual quantification of 41 target phenolic compounds was carried out by liquid chromatography-tandem mass spectrometry (LC-MS/MS). Other indexes such as the total phenolic content (TPC) and antioxidant activity (AA) were also evaluated. Finally, advanced statistical tools such as analysis of variance (ANOVA) and principal component analysis (PCA) were employed to obtain models that allow classifying the different honeys according to their origins.

## 2. Materials and Methods

### 2.1. Chemicals, Reagents and Materials

The target phenolic compounds, their CAS numbers, molecular mass, log Kow, retention time and MS/MS transitions are summarized in Table 1. Methanol and ultrapure water, both MS grade, were supplied by Scharlab (Barcelona, Spain). Hydrochloric acid, formic acid, Folin–Ciocalteu’s phenol reagent (2M), 2,2-diphenyil-1-picrylhydrazyl (DPPH), and 6-hydroxy-2,5,7,8-tetramethylchroman-2-carboxylic acid (Trolox^®^) were purchased from Sigma–Aldrich (Darmstadt, Germany). Sodium carbonate was supplied by Panreac (Barcelona, Spain).

Phenolic individual standard stock solutions (500–1000 µg mL^−1^) were prepared in methanol. Further dilutions and mixtures were prepared in acidified water (0.1% formic acid)/methanol (80:20, *v*/*v*) (AW/MeOH). All solutions were stored at −20 °C and protected from light. All chemicals and reagents were of analytical grade.

A vortex stirrer by Velp Scientifica (Usmate, Italy) and an ultrasound bath (50 kHz) from JP Selecta (Barcelona, Spain) were employed to perform the extractions.

### 2.2. Honey Samples

Ninety-one honey samples from Galicia (Northwest Spain) were kindly supplied by the protected geographical indication (P.G.I.) Mel de Galicia. Samples were received in glass jars sealed with aluminum caps. They were stored in the original containers at controlled temperature (15 °C) and kept away from light until their analysis.

The methodology used for the study of the botanical characteristics was based on the determination of the pollen contained in the honey by centrifugation. In total, 52% of the honey samples contained between 2000 and 10,000 grains of pollen per gram of honey, according to the classes of Maurizio; 39% of these samples contained between 10,000 and 50,000 grains of pollen per gram of honey [19]. The pollen spectrum of the samples consisted of 82 different pollen types, with 45% of them likely to be labelled as monofloral, while the remaining 55% were considered multi-floral, in which was included 16% whose majority origin was honeydew (HD). As regards monofloral honeys, the chestnut (CN, 34%), the blackberry (BL, 27.3%), the eucalyptus (EU, 25%) and, to a lesser extent, the heather (HE, 13.7%) stand out.

It should be noted that, as for the main proportion of the honey produced in Galicia, the main types were *Castanea, Eucalyptus, Erica, Rubus and Cytisus*, all of them in the dominant category or as companion in the pollen spectrum of honey.

### 2.3. VE-UAE Procedure

**Figure 1:**
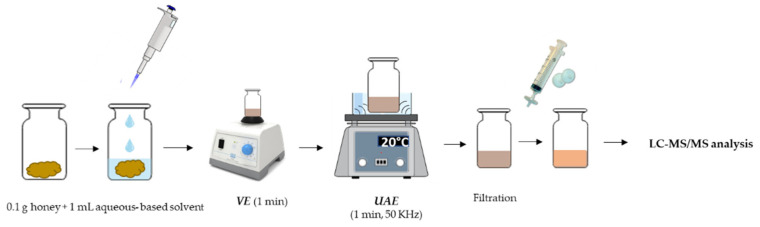
Schematic representation of the VE-UAE experimental procedure.

Under the optimal experimental conditions (see Section 3.2), 0.1 g of honey were weighted in a 1.8 mL glass vial and 1 mL of acidified water (0.1% formic acid)/methanol (80:20, *v*/*v*) (AW/MeOH) was added. The vial was sealed with an aluminum cap furnished with PTFE-faced septa and the solution was stirred by vortex for 1 min. Afterwards, the vial was immersed in an ultrasound bath for 1 min (20 °C, 50 KHz). The obtained extract was filtered through 0.22 μm polytetrafluoroethylene (PTFE) filters and directly injected in the LC-MS/MS system for phenols analysis (see Section 2.6). The experimental procedure is summarized in Figure 1.

### 2.4. Determination of TPC

The total phenolic content (TPC) of honey samples was determined according to the Folin–Ciocalteu (FC) colorimetric method described by Singleton and Rosssi [23]. Honey sample preparation was performed employing a modified method of Pauliuc et al. [16]. Briefly, 0.5 g of honey sample were diluted in 5 mL of methanol/water (40:60, *v*/*v*, pH = 2, HCl) and magnetically stirred for 15 min. Afterwards, 1.3 mL of this solution was diluted (1:10, *v*/*v*) in water up to a final volume of 13 mL. Then, an aliquot of 5 mL was placed on a Falcon tube and 100 μL of Folin–Ciocalteu’s phenol reagent and 1 mL of Na_2_CO_3_ solution (20%, w/v) were added. The Falcon tubes were kept away from light for 30 min. Afterwards, the absorbance was measured at 760 nm in a UV-Vis spectrophotometer Shimadzu UVmini-1240 (Kyoto, Japan). The TPC was quantified employing a calibration curve prepared with gallic acid standards solutions ranging between 1–20 mg L^−1^ (R^2^ = 0.9990) and expressed as mg of gallic acid equivalent (GAE) per 100 g of honey (mg GAE 100 g^−1^).

### 2.5. Determination of AA

The antioxidant activity (AA) was determined by a modified method of Brand–Williams et al. [24]. Briefly, 200 μL of the honey solution (0.5 g of honey diluted in 5 mL of methanol/water, 40:60, *v*/*v*, pH = 2, HCl) were introduced in a Falcon tube and 3.9 mL of the DPPH reagent solution (0.1 mM in methanol) were added. After 30 min in the absence of light, the absorbance was measured at 515 nm. The AA was quantified employing a calibration curve prepared with Trolox^®^ (0.1–0.9 mmol TRE g^−1^, R^2^ = 0.9970). The AA were expressed as micromoles of Trolox^®^ equivalents (TRE) per 100 g of honey (µmol TRE 100 g^−1^).

### 2.6. LC-MS/MS Analysis

The optimal instrumental conditions for the detection of the target phenols were adapted from Celeiro et al. [25]. LC-MS/MS analysis was performed employing a Thermo Scientific (San José, CA, USA) instrument based on a TSQ Quantum Ultra^TM^ triple quadrupole mass spectrometer equipped with a HESI-II (heated electrospray ionization) source and an Accela Open autosampler with a 20 μL loop. The chromatographic separation was achieved on a Kinetex C18 column (2.6 μm, 100 × 2.1 mm) with a guard column (SecurityGuard^TM^ ULTRA Holder) obtained from Phenomenex (Torrance, CA, USA). The injection volume was 10 μL and the column temperature was set at 50 °C. The mobile phase consisted of water (A) and methanol (B), both containing 0.1% formic acid. The eluted program started with 5% of B (held 5 min), it was up to 90% of B over 11 min (held 3 min). Then, initial conditions were reached in 5 min. The mobile phase flow rate was 200 μL min^−1^. The total run time for each injection was 20 min. The mass spectrometer and the HESI-II source were working simultaneously in the positive and negative mode (see ionization mode for each target compound in Table 1). Selected reaction monitoring (SRM) acquisition mode was implemented monitoring 2 or 3 transitions per compound (see Table 1), for an unequivocal identification and quantification of the target compounds. The system was operated by Xcalibur 2.2 and Trace Finder^TM^ 3.2.

### 2.7. Statistical Analysis

Analysis of variance (ANOVA) and principal component analysis (PCA) were performed employing Statgraphics Centurion XVIII software package (Manugistics, Rockville, MD, USA).

## 3. Results

### 3.1. Selection of the Solvent

The 41 target phenols present a high polarity range, as can be seen in Table 1, with log K_OW_ values ranging between 0.2 and 3.5. Therefore, their chromatographic separation and response are expected to be highly dependent on the dilution solvent. Different aqueous- based solvents were tested since one of the objectives of this work is the development of a green methodology, reduced usage of toxic solvents.

**Figure 2:**
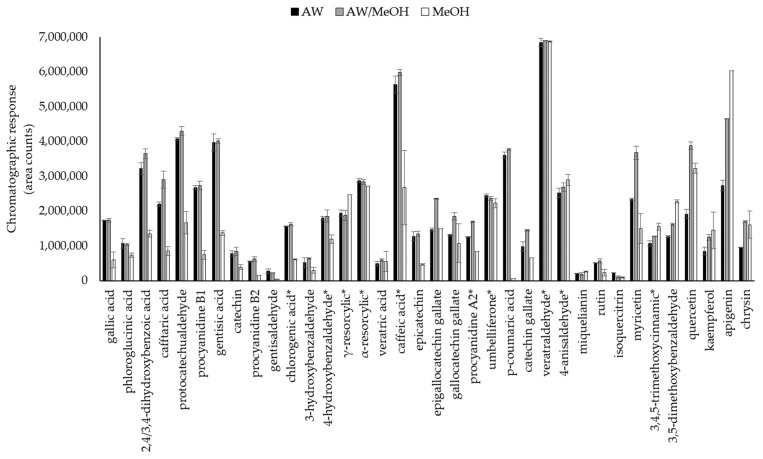
Chromatographic response for some phenols standard solutions (200 μg L^−1^) prepared in AW: acidified water (0.1% formic acid); AW/MeOH: acidified water (0.1% formic)/methanol (80:20, *v*/*v*); MeOH: methanol. * Divided/10.

Experiments were performed employing standard solutions containing the 41 target phenols at 200 μg L^−1^ prepared in: methanol (MeOH), acidified water with 0.1% formic acid (AW) and acidified water (0.1% formic acid)/methanol (80:20, *v*/*v*) (AW/MeOH). Results for some target phenols from high polar to low polar ones are shown in Figure 2. As can be seen, both aqueous based- solvents provided the highest chromatographic response for most compounds, especially for the highest polar ones, such as cinnamic- and benzoic- acids derivatives (gallic-, caftaric-, gentisic-, chlorogenic- acid, etc.). In contrast, the use of methanol to prepare the standard solutions for these compounds, resulted in chromatographic responses up to three times lower than those obtained with the aqueous-based solvents. Regarding medium polarity compounds, lower differences were observed for some compounds (α-resorcylic acid, umbelliferone, veratraldehyde, etc.) between the responses for the three tested solvents, whereas others achieved worse response for MeOH (epicatechin, p-coumaric acid, etc.). On the other hand, higher responses were obtained with AW/MeOH and MeOH for the low polar compounds, such as the flavonols quercetin and kaempferol and the flavones apigenin and chrysin.

**Figure 3:**
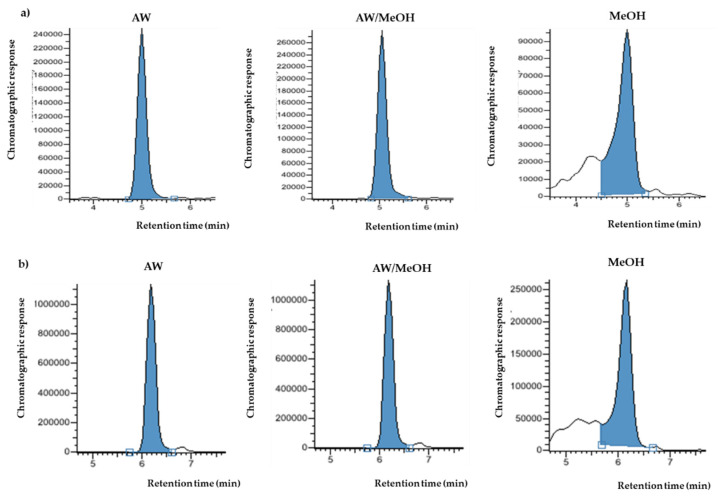
Peak shape comparison standard solutions (200 μg L^−1^) prepared in AW, AW/MeOH and MeOH for: (**a**) protocatechualdehyde; (**b**) chlorogenic acid.

As it is well known, the solvent not only affects the chromatographic response (abundance) of the analytes, but also highly affects the retention efficiency and thus, the chromatographic peak shape. Figure 3 shows the comparison between the chromatographic peaks for protocatechualdehyde (Figure 3a) and chlorogenic acid (Figure 3b) (200 μg L^−1^) prepared in AW, AW/MeOH and MeOH.

As can be seen, the standard prepared in methanol presented the worst peak shape, whereas standard prepared in AW and AW/MeOH showed in both cases satisfactory peak resolution. This behaviour was similar for most compounds, especially for those eluting first. Therefore, in view of these results, both aqueous based-solvent solutions were selected for further experiments.

### 3.2. VE-UAE Optimization

As previously commented, honey is a viscous and complex matrix, which makes not easy work with. Therefore, the selection of the most suitable extraction solvent is crucial to obtain the highest extraction efficiency. In this case, the extraction solvent and sample amount were optimized to obtain not only the highest extraction efficiency, but also to assess the possibility of miniaturizing the sample preparation procedure, fulfilling with the green chemistry principles.

#### 3.2.1. Extraction Solvent

**Figure 4:**
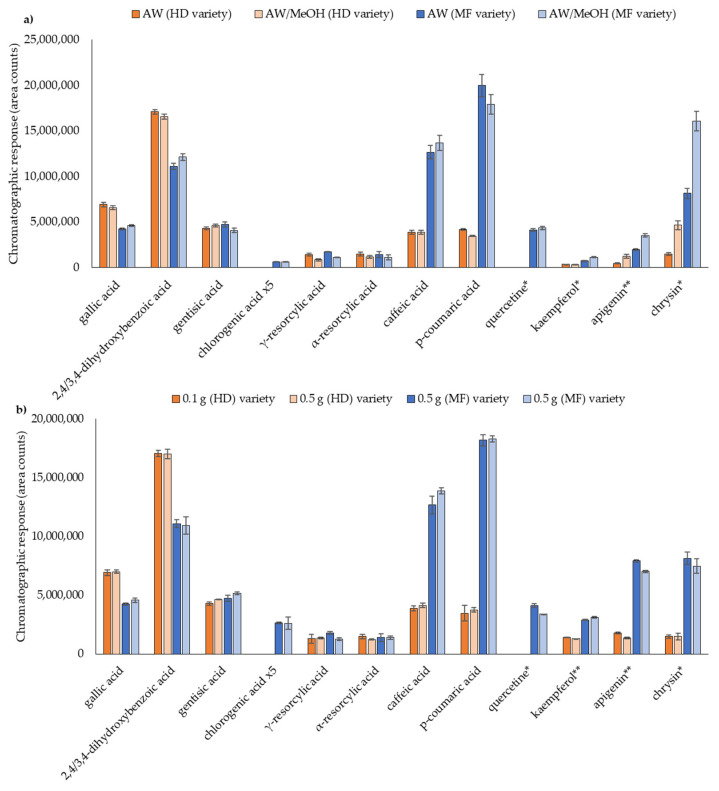
Optimization of the (**a**) extraction solvent; (**b**) sample size for two honey varieties (HD10 and MF32). * Response multiplied ×5; ** multiplied ×20.

Both aqueous-based solvents pre-selected in the preliminary studies, AW and AW/MeOH (see Section 3.1), were employed to prepare the honey samples. In this case, 0.1 g of honey in 1 mL of solvent were employed. Results for some detected compounds in two different honey samples from different origin, honeydew (HD) and multi-floral (MF) are represented in Figure 4a. In general, responses were similar for both aqueous- based solvents, excluding kaempferol, apigenin and chrysin. For these compounds, higher chromatographic responses, up to two times, were obtained in the two honey varieties, when AW/MeOH was employed as extractant. These results are in concordance with those previously obtained for the standard solutions of these flavones derivatives. For this reason, the solution AW/MeOH was selected.

#### 3.2.2. Sample Amount

Until now, in most honey studies the employed amount of sample usually involves the use of several grams of honey [16,20,26]. Since one of the objectives of the work is to obtain a miniaturized methodology with a low sample, reagents and solvents consumption, two different sample sizes were evaluated: 0.1 g and 0.5 g diluted 1:10 w/v. Results are depicted in Figure 4b, for two honey samples varieties, HD and MF. As can be seen, in all cases responses were similar employing 0.1 g and 0.5 g, concluding that the use of only 0.1 g of honey were representative and homogeneous. Therefore, 0.1 g of honey sample and 1 mL of AW/MeOH were selected, allowing a miniaturization of the extraction VE-UAE procedure.

### 3.3. VE-UAE-LC-MS/MS Performance

Under the optimal experimental conditions that involve the use of only 0.1 g of honey sample diluted in 1 mL of AW/MeOH, the whole VE-UAE-LC-MS/MS method was validated in terms of linearity, accuracy and precision. Limits of detection (LODs) were also calculated. Results are summarized in Table 2. Calibration curves were prepared in AW/MeOH containing the 41 target phenolic compounds, covering a concentration range for most compounds from 5 to 10,000 ng L^−1^, with 11 concentration levels (5, 10, 20, 50, 100, 200, 500, 1000, 2000, 5000 and 10,000 ng L^−1^) and three replicates per level. The method showed a good linearity, with coefficients of determination (R^2^) higher than 0.99. Instrumental precision was evaluated within a day (*n* = 4) and amongst days (*n* = 5) for all the calibration concentration levels. Relative standard deviation (RSD) values for 200 μg L ^−1^ are shown in Table 2, with mean values about 10%. To assess the accuracy of the proposed method, recovery studies were carried out employing a multi-floral honey sample (MF28). It is worth noting that only a few methods demonstrated accuracy for such a high number of phenolic compounds in honey samples and most of them imply the use of artificial matrices [20] or further experimental steps, mainly based on solid-phase extraction (SPE) to remove matrix components such as sugars [27]. The study was performed spiking the honey sample with the 41 target phenolic compounds at 2 μg g^−1^. Results depicted in Table 2 show that recovery values ranged between 70 and 100% for most compounds with RSD values lower than 8%. Recovery percentages obtained in other studies that apply UAE were higher for some phenolic acids (gallic acid, p-coumaric acid) while higher values were obtained in the present study for other compounds, such as myricetin and kaempferol. The degradation of some flavonoids during the extraction procedure assisted with irradiation was demonstrated [20]. Those undesirable effects were not observed in the present study since UAE was only applied for 1 min.

Limits of detection (LODs) were calculated as the compound concentration giving a signal-to-noise ratio of three (S/N = 3) employing the honey sample spiked with the target compounds. Results depicted in Table 2 show that they were at the ng g^−1^ level for all target phenolic compounds.

### 3.4. Analysis of Real Honey Samples

#### 3.4.1. TPC and AA

TPC and AA results for the 91 analyzed samples are summarized in Tables S1 and S2 for samples collected in 2018 and 2019, respectively. The ranges, mean and median concentrations for TPC, AA and individual phenolic compounds are shown in Table 3.

Results for TPC were similar in the two evaluated seasons. As shown in Table 3, TPC values ranged between 48–203 mg GAE 100g^−1^. As expected, a relationship seems to exist between the total concentration of target phenolic compounds and the TPC values, since the highest TPC was found in the heather sample HE1 that shows the highest sum of phenolic compounds, 252 μg g^−1^. On the other hand, most of EU and BL honey samples achieved low TPC.

The AA index ranged between 15–1017 μmol TRE 100 g^−1^ for the two seasons. Samples of honeydew achieved the highest antioxidant activity, reaching 1006 and 1017 μmol TRE 100 g^−1^ in sample HD4 and HD11, respectively. Results are in concordance, since most honey samples with high TPC values achieved high AA, as well. In the same way, the honey sample (EU11) with the lowest TPC (48 mg GAE 100 g^−1^) also reached the minimum AA concentration (15 μmol TRE 100 g^−1^).

Results of TPC and AA obtained in the Galician honeys were in consonance with those reported in other honeys from the same and different origin [19,28]. Thus, both indexes do not allow differentiating Galician honeys from other honeys, although they might allow to distinguish between the different honey varieties (see also Section 3.5).

#### 3.4.2. Individual Phenolic Content

Individual target phenolic compounds concentrations, as well as the sum of them for the 91 analyzed samples are summarized in Tables S1 and S2, and concentration ranges for the analyzed varieties are shown in Table 3.

Among the 41 target phenolic compounds, 22 were found in the samples of the 2018 season whereas 25 were detected in samples of 2019. The highest concentration of individual phenolic compounds was found in the heather variety (HE), with total phenolic compounds concentrations reaching 252 μg g^−1^, especially owing to the high content of 3-hydroxyphenylacetic acid (242 μg g^−1^ in sample HE1). The sum of phenolic compounds was highly influenced by the concentration of this compound since it was found in 33 of the 91 analyzed samples at a mean value of 35 μg g^−1^ and in the range 0.41–242 μg g^−1^. It is worth noting that these high 3-phenoxyphenylacetic acid contents do not confer high antioxidant activities to the HE honey, compared with those containing honeydew.

Regarding those samples that were not highly affected by 3-phenoxyphenylacetic acid, honeydew honeys (HD) contained high concentration of the sum of phenolic compounds, with 35 and 25 μg g^−1^ in 2018 and 2019 seasons, respectively. In the same way, the mixture chestnut/honeydew variety (CN/HD) reached concentrations up to 30 μg g^−1^ for the sum of the target phenolic compounds. On the other hand, the lowest concentration for the sum of phenolic compound was detected in a eucalyptus honey (EU11) with 3.4 μg g^−1^. In general, results were similar in both seasons and the values were in concordance with the TPC and AA.

The most abundant phenolic compound, detected in all the analyzed honey samples, was p-hydroxybenzoic acid, in a concentration range from 0.68 to 5.1 μg g^−1^. Other benzoic- and hydroxycinnamic- derivates acids, such as gallic or protocatechuicacid, were found at high concentrations, up to 10 μg g^−1^ in honeydew (HD), 8.0 μg g^−1^ in chestnut/honeydew (CN/HD) and 4.6 μg g^−1^ in blackberry (BL) varieties. It is important to note that 7 of the 14 chestnut samples collected during the 2019 campaign contained honeydew, which could contribute to the concentration increase. In HD samples, gentisic acid (present in 86 honeys) reached the highest mean concentration of 2.7 μg g^−1^. Additionally, p-coumaric acid, which was found in all samples, achieved concentrations up to 12 μg g^−1^ in multi-floral (MF) honeys. P-coumaric acid content fluctuations could be observed within the same variety; however, the highest concentrations and fluctuations occurred in multi-floral (MF) honeys, with values ranging from 0.1 to 12 µg g^-1^.

Veratric acid was detected only in six honey samples of several types (BL, CN, HD and MF) at concentrations around 1.5 μg g^−1^.

As regards aldehydes, 3-hydroxybenzaldehyde and 4-hydroxybenzaldehyde appeared in several samples of different honey varieties (EU, BL HE, CN and MF) at concentrations of up to 1.2 and 2.0 μg g^−1^, respectively. It is important to note the absence of these two aldehydes in the honeydew samples as well as in the chestnut honeys with honeydew (CN/HD), except in sample HD10 in which 3-hydroxybenzaldehyde appeared at a concentration of 0.10 μg g^−1^.

Concerning other families, 2 of the 3 targeted flavonols were found in the samples: quercetin in 69 samples and kaempferol in 40 honey samples, whereas myricetin was not detected in any sample. Additionally, the flavones chrysin and apigenin were found in 91 and 84 honey samples, respectively. They were present at concentrations up to 5.9 μg g^−1^, for chrysin in BL8, and 0.43 μg g^−1^, for apigenin in MF5. In contrast, flavanol compounds (catechin, epicatechin, gallocatechin gallate) were not detected in the analyzed samples.

### 3.5. Chemometric Study

#### 3.5.1. Analysis of Variance (ANOVA)

One way ANOVA was performed to assess statistical differences between the botanical origin of honeys based on their bioactive properties (TPC and AA) and their phenolic profile/composition at a 95% of confidence level. One way ANOVA was selected instead of two ways because the harvest year was not statistically significant (data not shown).

**Figure 5:**
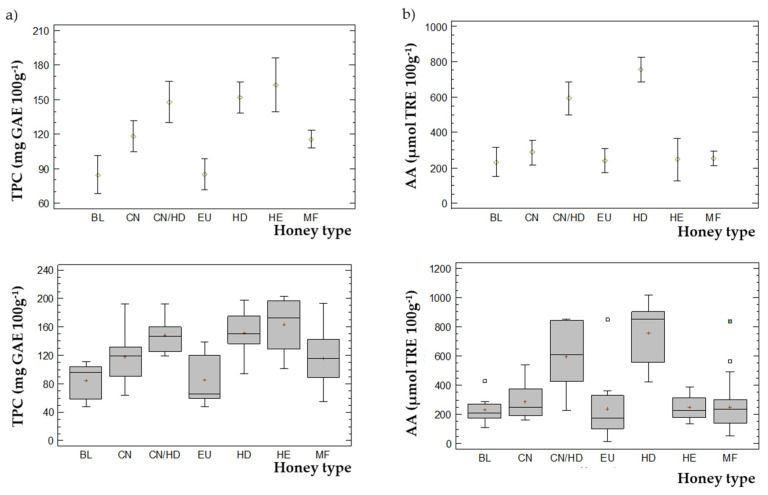
Mean values and box-and-whiskers plots for the different honey varieties based upon (**a**) TPC; (**b**) AA.

The mean values and box-and-whiskers plots obtained from TPC and AA values are depicted in Figure 5a,b, respectively.

Concerning TPC, three different homogeneous groups could be determined and be easily visualized in Figure 5a. One group was formed by BL and EU honey varieties, the second one was formed by CN and MF, and the last group was formed by CN/HD, HD and HE honeys. These results were confirmed with those obtained in a multiple range least significant difference (LSD) test (data not shown).

Regarding AA (Figure 5b), three statistically different groups were obtained. The first one formed by HD, the second by the mixture CN/HD and the rest of honey varieties (BL, CN, EU, HE and MF) constituted the third one.

**Figure 6:**
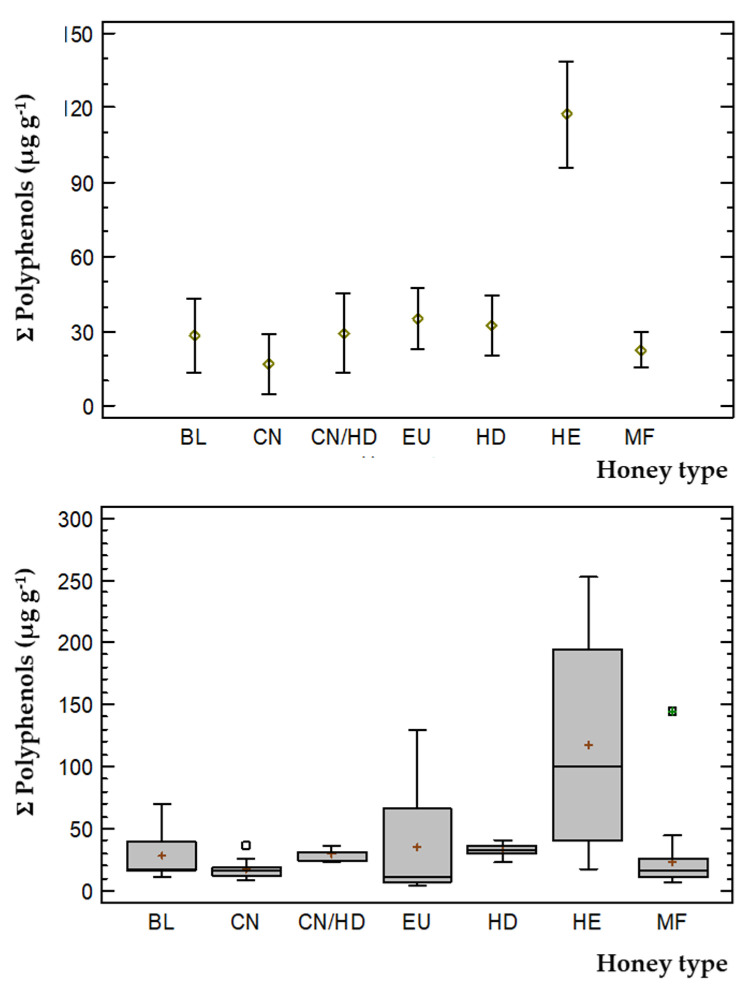
Mean values and box-and-whiskers plots for the different honey varieties based upon the sum of target phenolic compounds concentration.

Considering the total concentration of phenolic compounds found in the analyzed samples (see Figure 6), only two groups could be differentiated. This was in concordance with the multiple range test: one formed by HE honey variety, and another group formed by the other varieties (BL, CN, CN/HD, EU, HD and MF). These results demonstrate the high influence of the concentration of 3-hydroxyphenylacetic acid in the sum of phenolic compounds, since it was detected at concentrations over to 200 μg g^−1^ in the HE samples, as already mentioned in Section 3.4.2.

#### 3.5.2. Principal Components Analysis (PCA)

Honey classification based upon the presence of different target phenolic compounds was one of the objectives of this study. For this reason, a principal components analysis (PCA) was employed by means of a data matrix including the 91 analyzed samples and 25 variables given by the responses of the 25 phenolic compounds detected after LC-MS/MS analysis.

**Figure 7:**
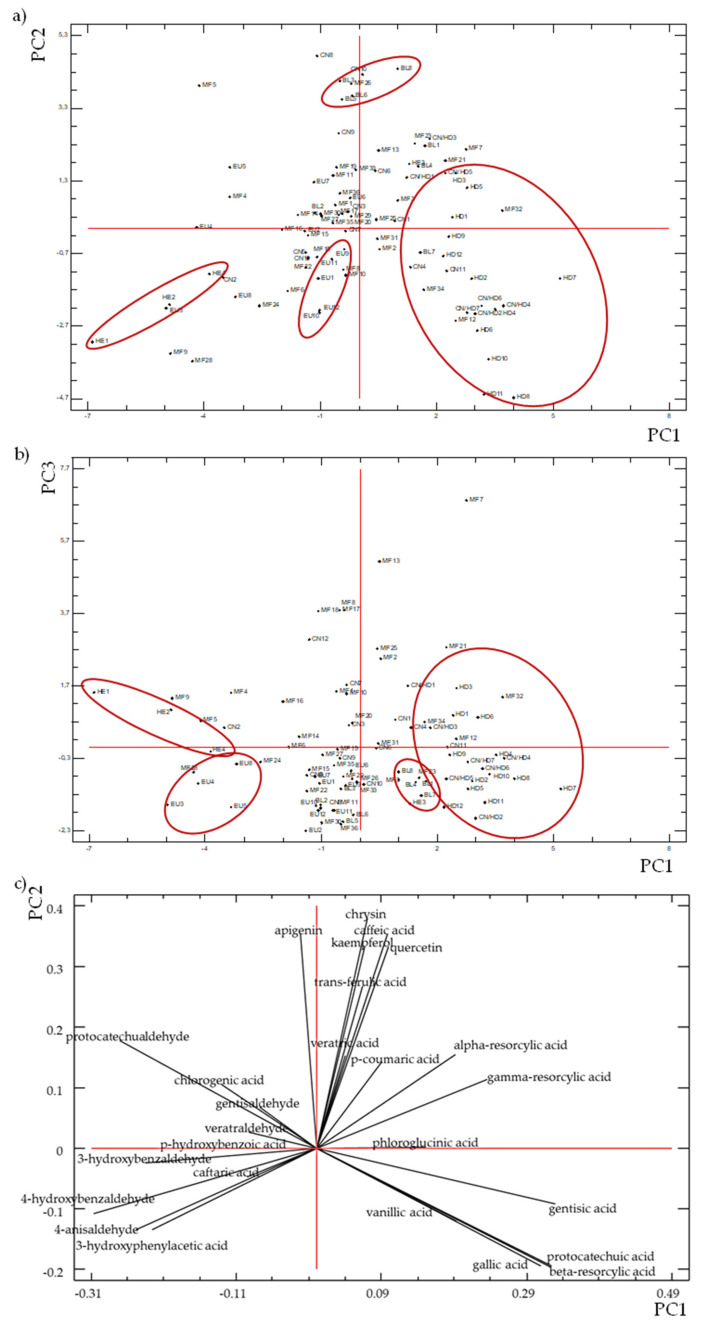
PCA analysis. Scatter plot of (**a**) PC1 and PC2; (**b**) PC1 and PC3; (**c**) plot of component weights for PC1 and PC2 of the 91 analyzed samples. HE: heather, HD: honeydew, CN: chestnut, CN/HD: chestnut/honeydew, EU: eucalyptus, MF: multi-floral, BL: blackberry.

The phenolic compounds responses were auto-standardized by the Statgraphics software. Only principal components with the largest eigenvalues and greater than one were retained (Kaiser criterion). Six principal components (PC) were then retained and were enough to explain about 70% of variance (data not shown). As an example, the PC1 and PC2 and the PC1 and PC3 scatter plots for the 91 samples of different honey varieties are depicted in Figure 7a,b, respectively. A plot of component weights for PC1 and PC2 is also depicted in Figure 7c.

PC1 was mainly positively influenced by acids (gallic acid, β-resorcylic acid, protocatechuic acid and gentisic acid), and negatively by aldehydes (protocatechualdehyde, 3-hydroxybenzaldehyde, 4-hydroxybenzaldehyde and 4-anisaldehyde) (Figure 7c).

In contrast, PC2 was highly positively affected by phenolic acids (caffeic acid, *trans*-ferulic acid and p-coumaric acid), flavones (apigenin and chrysin), flavonols (quercetin and kaempferol) (Figure 7c).

As can be seen in Figure 7a,b, three different groups can be distinguished. Honeydew (HD) honeys as well as chestnut with honeydew (CN/HD) can be classified as one group positively highly influenced by PC1. On the other hand, three samples of heather honey (HE), negatively affected by PC1, can be gathered, whereas BL honeys are clearly differentiated according to PC2. Besides, a group including some of the EU honey samples can be differentiated.

Chestnut honeys are not clearly differentiated by any of the PC, although four of them (CN6, CN8, CN9 and CN10) show a simultaneous high concentration of quercetin, chrysin and *trans*-ferulic acid, as expressed in PC2 (Figure 7a). Blackberry honeys also contain high proportions of these three compounds plus kaempferol.

The plot of component weights depicted in Figure 7c indicates which compounds are dominant for each type of honey. For honeydew honey, gallic acid is the main chemical marker along with β-resorcylic acid and protocatechuic acid.

In the case of heather honeys, 4-anisaldehyde, 3-hydroxyphenylacetic acid and 4-hydroxybenzaldehyde appear as main markers.

Most of the 32 multi-floral honeys are located at the centre of the PCA-2D component plots, confirming that with such a mixture of nectars coming from multiple plant species, no specific group and no specific origin can be identified.

Nevertheless, these results also show that PCA is a suitable approach to identify groups of honey from different botanical origins.

## 4. Conclusions

91 Galician honeys obtained from different botanical origins and nectar sources were analyzed to assess their similarities, differences and correlations in terms of phenolic profiles. A miniaturized, fast and environmentally friendly methodology based on VE-UAE-LC-MS/MS was successfully developed. Results revealed the presence of 25 out of the 41 target phenolic compounds in the 91 analyzed samples. TPC and AA were also evaluated, showing mean values around 121 mg GAE 100g^−1^ of honey and 340 μmol TRE 100g^−1^, respectively. ANOVA and PCA results based on TPC, AA and ∑phenolic compounds concentrations, revealed significant differences depending on the honey variety, demonstrating that phenolic compounds can be used as indicators to identify their floral origin. This study proves that the combination of chromatographic analysis with mass spectrometry detection and PCA are suitable tools to investigate the botanical authentication of honey and to guarantee its quality and origin.

## Figures and Tables

**Table 1:** Target phenolic compounds: CAS number, molecular mass (Mm), log Kow, retention time, ionization mode and MS/MS transitions.

Phenolic Compounds	CAS	Mm (g mol^−1^)	log Kow	Retention Time (min)	Ionization Mode ^1^	MS/MS Transitions ^2^
gallic acid	149-91-7	170.1	0.70	2.61	−	169.02 → 125.04 (17) 169.02 → 153.1 (15)
phloroglucinic acid	71989-93-0	188.1	1.28	4.22	+	168.98 → 150.99 (17) 168.98 → 83.02 (23) 168.98 → 107.02 (22)
β-resorcylic acid ^3^	89-86-1	154.1	1.63	5.00	+	153.00 → 109.05 (16) 153.00 → 65.09 (19) 153.00 → 67.07 (23)
protocatechuic acid ^3^	99-50-3	154.1	0.86	5.00	+	152.98 → 109.04 (17) 152.98 → 91.04 (28) 152.98 → 108.03 (26)
caftaric acid	67879-58-7	312.2	0.21	4.78	−	310.96→ 178.97 (17) 310.96 → 148.96 (14)
protocatechualdehyde	139-85-5	138.1	1.09	5.05	+	137.07 → 136.11 (21) 137.07 → 91.09 (24) 137.07 → 92.13 (25)
procyanidin B1	20315-25-7	578.5		5.07	−	577.03 → 407.06 (26) 577.03 → 288.93 (25) 577.03 → 424.97 (26)
p-hydroxybenzoic acid	99-96-7	138.1	1.58	5.35	−	137.00 → 93.00 (17) 137.00 → 65.00 (27)
gentisic acid	490-79-9	117.1	1.74	5.38	+	152.96 → 108.00 (24) 152.96 → 81.02 (21) 152.96 → 109.01 (16)
catechin	18829-70-4	290.3	0.51	5.50	+	289.00 → 245.02 (17) 289.00 → 203.11 (22)
3-hydroxyphenylacetic acid	621-37-4	152.2	0.85	5.70	−	151.00 → 65.00 (20) 151.00 → 79.00 (20)
procyanidin B2	29106-49-8	578.5	2.29	5.96	−	577.03 → 407.06 (26) 577.03 → 288.93 (25) 577.03 → 424.97 (26)
gentisaldehyde	1194-98-5	138.1	1.53	6.11	+	136.99 → 108.02 (21) 136.99 → 81.08 (18) 136.99 → 109.04 (14)
chlorogenic acid	327-97-9	354.3	1.01	6.12	+	353.00 → 191.07 (22) 353.00 → 85.09 (43) 353.00 → 93.07 (45)
3-hydroxybenzaldehyde	100-83-4	122.1	1.29	6.22	+	121.02 → 93.05 (20) 121.02 → 92.05 (23) 121.02 → 120.04 (19)
4-hydroxybenzaldehyde	123-08-0	122.1	1.36	6.25	+	122.97 → 95.05 (13) 122.97 → 51.10 (36) 122.97 → 77.05 (20)
vanillic acid	121-34-6	168.2	1.43	6.29	−	167.00 → 108.00 (27) 167.00 → 152.00 (18)
γ-resorcylic acid	303-07-1	154.1	2.20	6.33	+	153.00 → 109.05 (17) 153.00 → 65.09 (21) 153.00 → 135.02 (16)
α-resorcylic acid	99-10-5	154.1	0.86	6.33	+	152.97 → 109.01 (15) 152.97 → 65.06 (16) 152.97 → 67.05 (20)
veratric acid	93-07-2	182.2	1.61	6.45	+	182.96 → 137.08 (6) 182.96 → 106.99 (22)
caffeic acid	331-39-5	180.2	1.15	6.50	−	178.98 → 135.03 (19) 178.98 → 134.01 (28)
epicatechin	35323-91-2	290.3	0.51	6.56	+	289.00 → 245.02 (17) 289.00 → 203.11 (22)
epigallocatechin gallate	989-51-5	458.4	2.56	6.79	+	457.15 → 169.05 (21) 457.15 → 125.09 (42) 457.15 → 305.09 (21)
gallocatechin gallate	84650-60-2	458.4	2.56	7.29	+	457.15 → 169.05 (21) 457.15 → 125.09 (42) 457.15 → 305.09 (21)
procyanidin A2	41743-41-3	576.5	2.52	7.32	−	577.09 → 287.00 (32) 577.09 → 136.98 (62) 577.09 → 425.08 (13)
umbelliferone	93-35-6	162.1	1.58	7.80	+	162.99 → 107.04 (22) 162.99 → 77.05 (34) 162.99 → 91.05 (20)
p-coumaric acid	501-98-4	164.2	1.79	7.89	+	163.02 → 119.07 (18) 163.02→ 93.07 (37) 163.02 → 117.05 (38)
catechin gallate	130405-40-2	442.3	2.62	8.01	+	441.13 → 289.13 (20) 441.13 → 125.08 (42) 441.13 → 169.05 (24)
*trans*-ferulic acid	537-98-4	194.2	1.51	8.33	−	192.80 → 177.90 (12) 192.80 → 133.90 (16)
veratraldehyde	120-14-9	166.2	1.22	8.92	+	167.01 → 139.05 (13) 167.01 → 108.05 (21) 167.01 → 124.03 (18)
4-anisaldehyde	123-11-5	136.1	1.76	10.03	+	136.97 → 109.05 (12) 136.97 → 77.05 (23) 136.97 → 94.04 (18)
miquelianin	22688-79-5	478.4	0.20	10.32	+	479.09 → 461.50 (14) 479.09 → 302.96 (18)
rutin	153-18-4	610.5	0.15	10.35	−	609.18 → 270.92 (96) 609.18 → 178.87 (44) 609.18 → 300.01 (37)
isoquercitrin	482-35-9	463.4	0.76	10.43	+	465.07 → 256.90 (41) 465.07 → 302.97 (14)
myricetin	529-44-2	318.2	1.42	11.43	+	319.00 → 153.02 (31) 319.00 → 217.06 (31) 319.00 → 245.06 (27)
3,4,5-trimethoxycinnamic acid	90-50-6	238.2	1.58	11.59	+	239.03 → 221.04 (11) 239.03 → 162.99 (27) 239.03 → 190.01 (19)
3,5-dimethoxybenzaldehyde	7311-34-4	166.2	1.87	11.81	+	167.15 → 124.03 (17) 167.15 → 77.05 (26)
quercetin	117-39-5	302.2	1.48	12.10	+	303.09 → 229.10 (28) 303.09 → 153.04 (33)
kaempferol	520-18-3	286.2	1.96	12.57	−	285.07 → 184.91 (30) 285.07 → 239.12 (35)
apigenin	520-36-5	270.2	3.02	12.63	−	269.09 → 117.12 (37) 269.09 → 149.12 (26) 269.09 → 151.06 (26)
chrysin	480-40-0	254.2	3.52	13.24	+	253.13 → 143.18 (30) 253.13 → 63.20 (34) 253.13 → 145.16 (31)

^1^ “−”and “+” indicate negative and positive ionization modes, respectively. ^2^ Underlined MS/MS transition used for quantification purpose.^3^ Isomers: 2,4/3,4-dihydroxybenzoic acid.

**Table 2:** VE-UAE_LC-MS/MS performance: Linearity, precision, recoveries and LODs.

Phenolic Compounds	Linearity	Precision (RSD, %)		Recovery (%)	LOD (ng g^−1^)
	R^2^	Intra-Day (*n* = 4)	Inter-Day (*n* = 5)		
gallic acid	0.9985	5.3	3.5	58.3 ± 9.0	39
phloroglucinic acid	0.9996	10	9.1	84.3 ± 4.7	137
β-resorcylic acid ^1^	0.9954	11	7.2	78 ± 11	54
protocatechuic acid ^1^	0.9937	3.4	4.5		
caftaric acid	0.9973	2.0	9.9	101 ± 5	22
protocatechualdehyde	0.9906	8.6	14	75.3 ± 5.2	16
procyanidine B1	0.9993	18	16	78 ± 11	17
gentisicacid	0.9996	14	16	90.5 ± 7.3	20
catechin	0.9917	6.5	15	61 ± 12	46
procyanidine B2	0.9926	3.9	18	59.0 ± 5.1	18
gentisaldehyde	0.9993	5.2	15	69.2 ± 1.0	91
chlorogenic acid	0.9992	4.3	2.6	59.2 ± 2.7	7.1
3-hydroxybenzaldehyde	0.9986	8.2	20	71.7 ± 4.4	17
4-hydroxybenzaldehyde	0.9978	13	15	113 ± 2	35
γ-resorcylic acid	0.9949	1.2	4.7	141 ± 2	14
α-resorcylic acid	0.9903	7.7	5.3	133 ± 7	12
veratric acid	0.9988	16	14	80.5 ± 4.6	40
caffeic acid	0.9962	4.3	5.6	76.1 ± 0.8	8.8
epicatechin	0.9946	11	9.7	54.7 ± 0.6	6.9
epigallocatechin gallate	0.9940	18	16	76.1 ± 8.0	121
gallocatechin gallate	0.9990	17	3.9	45.1 ± 8.3	79
procyanidine A2	0.9975	1.6	6.4	83.8 ± 4.2	12
umbelliferone	0.9928	8.9	12	106 ± 1	8.0
p-coumaric acid	0.9980	2.6	6.8	66.6 ± 1.7	7.9
catechin gallate	0.9988	7.9	11	71.7 ± 0.4	16
veratraldehyde	0.9999	4.2	4.1	77.3 ± 1.0	10
4-anisaldehyde	0.9953	5.2	9.1	88.9 ± 6.2	30
miquelianin	0.9928	13	6.1	115	192
rutin	0.9928	8.3	13	95.3 ± 6.7	29
isoquercitrin	0.9915	15	2.1	-	163
myricetin	0.9982	10	18	103 ± 19	216
3,4,5-trimethoxycinnamic acid	0.9944	12	12	86.9 ± 5.3	21
3,5-dimethoxybenzaldehyde	0.9985	15	13	76.5 ± 9.5	156
quercetin	0.9992	10	19	-	39
kaempferol	0.9906	5.9	9.7	61.5 ± 5.9	45
apigenin	0.9168	7.5	5.4	53.8 ± 2.8	7.0
chrysin	0.9948	10	6.6	70.3 ± 13	55
*trans*-ferulic acid	0.9972	7.5	4.2	105 ± 2.2	41
vanillic acid	0.9980	2.1	5.5	87.0 ± 5.7	24
p-hydroxybenzoic acid	0.9922	9.8	4.9	n.c.	7.0
3-hydroxyphenylacetic acid	0.9977	7.1	4.4	n.c.	60

^1^ Sum of both isomers: 2,4/3,4- dihydroxybenzoic acid; n.c. Not calculated since the concentration in the sample was higher than the spike level (see sample MF28 in Table S2c).

**Table 3:** Range, mean concentration and median of phenolic compounds (μg g^−1^), TPC (mg GAE 100g^−1^) and AA (μmol TRE 100g^−1^) for eucalyptus (EU), blackberry (BL), chestnut (CN) and chestnut/honeydew (CN/HD) honeys, honeydew (HD), heather (HE) and multi-floral (MF) honeys.

Phenolic Compounds	EU (*N* = 12)				BL (*N* = 8)				CN (*N* = 12)				CN/HD (*N* = 7)				HD (*N* = 12)				HE (*N* = 4)				MF (*N* = 36)			
	*N*	Range	Mean	Median	*N*	Range	Mean	Median	*N*	Range	Mean	Median	*N*	Range	Mean	Median	*N*	Range	Mean	Median	*N*	Range	Mean	Median	*N*	Range	Mean	Median
gallic acid	7	0.13–0.47	0.26	0.22	8	0.29–2.1	0.97	0.56	9	0.18–3.3	0.92	0.46	7	1.6–6.8	4.3	5.2	12	1.6–9.8	5.5	6.4	2	0.20–1.1	0.65	0.65	28	0.15–4.5	0.73	0.30
phloroglucinic acid	0				2	0.21–0.34	0.28	0.28	2	0.13–0.48	0.12	0.12	4	0.29–0.87	0.47	0.36	2	0.3–1.4	0.86	0.86	1	0.38	0.38	0.38	3	0.18–0.32	0.27	0.32
β-resorcylic acid/protocatechuic acid	12	0.22–0.55	0.40	0.41	8	0.42–4.6	1.9	1.5	12	0.11–0.13	1.5	0.74	7	2.5–8.0	5.5	6.1	12	3.3–10	7.0	6.8	4	0.36–2.5	0.95	0.49	36	0.26–11	1.5	0.72
caftaric acid	2	0.06–0.10	0.08	0.08	1	0.05	0.05	0.05	0				0				0				0				1	0.08	0.08	0.08
protocatechualdehyde	8	0.05–0.27	0.12	0.09	5	0.07–0.19	0.14	0.15	9	0.09–0.25	0.17	0.19	1	0.05	0.05	0.05	0				3	0.16–0.23	0.20	0.22	29	0.05–0.43	0.15	0.12
gentisic acid	11	0.07–0.44	0.17	0.11	8	0.10–1.3	0.69	0.70	12	0.16–2.0	0.90	0.80	7	1.7–3.0	2.4	2.6	12	1.6–3.8	2.7	2.7	4	0.12–0.98	0.36	0.18	32	0.06–5.2	1.2	0.87
gentisaldehyde	3	0.31–0.54	0.45	0.49	0				4	0.18–0.43	0.27	0.23	1	0.64	0.64	0.64	0				1	0.32	0.32	0.32	2	0.22–0.27	0.25	0.25
chlorogenic acid	5	0.05–0.10	0.09	0.10	6	0.05–0.09	0.07	0.07	10	0.05–0.18	0.08	0.07	2	0.06–0.15	0.11	0.11	4	0.06–0.10	0.08	0.08	3	0.05–0.12	0.09	0.11	25	0.05–0.19	0.10	0.08
3-hydroxybenzaldehyde	4	0.13–0.87	0.59	0.68	2	0.10–0.22	0.16	0.16	1	0.30	0.30	0.30	0				1	0.10	0.10	0.10	2	0.73–1.2	0.97	0.97	4	0.12–0.97	0.44	0.33
4-hydroxybenzaldehyde	4	1.2–1.3	1.3	1.3	1	0.28	0.28	0.28	4	0.33–1.5	0.71	0.51	0				0				3	1.2–1.8	1.5	1.7	9	0.10–2.0	0.82	0.32
γ-resorcylic acid	3	0.05–0.07	0.06	0.06	6	0.05–0.09	0.07	0.07	10	0.05–0.12	0.09	0.10	6	0.06–0.10	0.09	0.10	11	0.05–0.12	0.08	0.07	1	0.08	0.08	0.08	18	0.05–0.31	0.10	0.10
α-resorcylic acid	2	0.06–0.07	0.07	0.07	6	0.05–0.08	0.06	0.06	9	0.08–0.11	0.09	0.09	6	0.05–0.10	0.08	0.09	7	0.06–0.13	0.09	0.08	1	0.07	0.07	0.07	23	0.06–0.28	0.09	0.08
veratric acid	0				2	0.2–1.9	1.6	1.6	1	1.6	1.6	1.6	0				1	1.8	1.8	1.8	0				2	0.89–1.1	1.1	1.1
caffeic acid	12	0.07–0.37	0.20	0.17	8	0.35–0.81	0.55	0.57	12	0.06–0.85	0.38	0.30	7	0.22–0.62	0.37	0.33	12	0.07–0.57	0.28	0.30	4	0.12–0.42	0.22	0.17	36	0.05–0.64	0.27	0.26
p-coumaric acid	12	0.48–4.2	1.3	0.85	8	0.44–4.1	2.2	2.1	12	0.5–11	3.3	2.0	7	1.4–6.7	3.3	1.7	12	0.27–3.5	1.7	1.5	4	0.05–2.6	0.87	0.41	36	0.14–12	4.02	2.25
veratraldehyde	7	0.05–0.12	0.07	0.07	1	0.09	0.09	0.09	0				0				2	0.06–0.07	0.07	0.07	2	0.06–0.10	0.08	0.08	12	0.05–0.45	0.14	0.06
4-anisaldehyde	1	0.28	0.28	0.28	0				3	0.13–0.41	0.26	0.24	0				0				3	0.14–0.77	0.50	0.59	10	0.12–0.72	0.33	0.22
quercetin	7	0.25–0.80	0.51	0.51	7	0.25–1.4	0.83	1.02	10	0.22–1.3	0.46	0.38	5	0.39–0.62	0.46	0.42	11	0.16–0.63	0.35	0.31	1	0.79	0.79	0.79	28	0.29–1.1	0.56	0.51
kaempferol	3	0.23–0.44	0.31	0.25	6	0.25–0.49	0.40	0.45	3	0.27–0.60	0.44	0.44	4	0.24–0.41	0.34	0.35	7	0.22–0.38	0.28	0.26	2	0.25–0.31	0.28	0.28	15	0.22–0.71	0.34	0.33
apigenin	10	0.06–0.39	0.15	0.12	8	0.13–0.31	0.22	0.23	12	0.07–0.31	0.15	0.13	7	0.09–0.21	0.14	0.10	11	0.06–0.20	0.13	0.15	4	0.08–0.19	0.13	0.13	32	0.05–0.43	0.15	0.13
chrysin	12	0.50–3.4	1.9	1.9	8	2.2–5.9	3.8	3.4	12	0.53–4.5	2.8	2.9	7	1.7–4.2	2.6	2.2	12	0.94–4.4	2.6	2.5	4	1.8–3.1	2.3	2.1	36	0.51–5.9	2.63	2.40
*trans*-ferulic acid	11	0.48–0.91	0.69	0.69	8	0.62–1.7	1.1	1.2	8	0.36–2.0	0.93	0.90	7	0.42–1.0	0.72	0.74	10	0.27–0.96	0.6	0.7	4	0.15–1.1	0.48	0.34	23	0.19–1.5	0.64	0.64
p-hydroxybenzoic acid	12	0.77–2.3	1.4	1.3	8	0.68–2.3	1.4	1.4	12	1.2–3.4	2.0	1.8	7	1.1–2.5	1.8	1.7	12	0.83–2.9	1.6	1.6	4	1.4–4.0	2.7	2.6	36	0.77–5.1	2.3	2.1
3-hydroxyphenylacetic acid	5	12–123	66	61	3	3.7–63	32	29	3	2.8–12	7.5	8.0	2	1.7–9.5	5.6	5.6	7	0.41–11	4.2	2.0	3	54–242	140	125	10	1.3–138	24	9.9
vanillic acid	0				1	0.27	0.27	0.27	2	0.07–0.20	0.14	0.14	2	0.15–0.40	0.28	0.28	8	0.14–6.2	1.1	0.30	2	0.10–0.12	0.11	0.11	6	0.07–0.58	0.31	0.28
∑ [phenolic compounds]	12	3.4–130	35	11	8	11–71	27	16	12	7.1–31	16	15	7	21–30	25	23	12	18–35	26	26	4	15–252	116	99	36	6.5–144	21	15
TPC (mg GAE 100g^−1^)	12	48–139	85	66	8	48–111	85	96	12	64–192	118	119	7	119–192	148	147	12	95–197	152	151	4	102–203	163	173	36	55–193	116	116
AA (µmol TRE 100g^−1^)	12	15–846	239	176	8	111–428	232	211	12	140–540	265	210	7	228–854	592.43	611	12	420–1017	756	852	4	138–392	272	279	36	56–837	252	236

## Data Availability

Data are available within the present article and Supplementary Materials.

## Supplementary Materials

The following are available online at https://www.mdpi.com/article/10.3390/foods10112616/s1, Table S1: concentration (μg g^−1^) of target phenolic compounds, TPC (mg GAE 100g^-1^) and AA (μmol TRE 100g^−1^) for: (a) EU, BL, CN and HD; (b) HE and MF honeys in the 2018 season. Table S2: concentration (μg g^−1^) of target phenolic compounds, TPC (mg GAE 100g^−1^) and AA (μmol TRE 100g^−1^) for: (a) EU and BL; (b) CN, CN/HD and HD; (c) HE and MF honeys in the 2019 season.

## References

[1] Cianciosi D., Forbes-Hernández T.Y., Afrin S., Gasparrini M., Reboredo-Rodriguez P., Manna P.P., Zhang J., Lamas L.B., Flórez S.M., Toyos P.A. (2018). Phenolic Compounds in Honey and Their Associated Health Benefits: A Review. Molecules.

[2] Pyrzynska K., Biesaga M. (2009). Analysis of phenolic acids and flavonoids in honey. TrAC Trends Anal. Chem..

[3] Jerković I., Tuberso C.I., Gugic M., Bubalo D. (2010). Composition of Sulla (Hedysarum coronarium L.) Honey Solvent Extractives Determined by GC/MS: Norisoprenoids and Other Volatile Organic Compounds. Molecules.

[4] Fakhlaei R., Selamat J., Khatib A., Razis A.F.A., Sukor R., Ahmad S., Babadi A.A. (2020). The Toxic Impact of Honey Adulteration: A Review. Foods.

[5] Codex Alimentarius Commission Codex Alimentarius Commission Standards.

[6] European Commission (2001). Directive 2001/110/EC of 20 December relating honey. Off. J. EC.

[7] (2014). Directive 2014/63/EU of the European parliament and of the council of 15 May 2014 amending council directive 2001/110/EC relating to honey. Off. J. EU.

[8] Thrasyvoulou A., Tananaki C., Goras G., Karazafiris E., Dimou M., Liolios V., Kanelis D., Gounari S. (2018). Legislation of honey criteria and standards. J. Apic. Res..

[9] Kečkeš S., Gašić U., Velickovic T.C., Milojković-Opsenica D., Natić M., Tešic Z. (2013). The determination of phenolic profiles of Serbian unifloral honeys using ultra-high-performance liquid chromatography/high resolution accurate mass spectrometry. Food Chem..

[10] Gašić U., Milojković-Opsenica D.M., Tešić Ž.L. (2017). Polyphenols as Possible Markers of Botanical Origin of Honey. J. AOAC Int..

[11] Jaganathan S.K., Mandal M. (2009). Antiproliferative Effects of Honey and of Its Polyphenols: A Review. J. Biomed. Biotechnol..

[12] Khalil M.L., Sulaiman S.A. (2010). The potential role of honey and its polyphenols in preventing heart disease: A review. Afr. J. Tradit. Complement. Altern. Med..

[13] Hossen M.S., Ali M.Y., Jahurul M.H.A., Abdel-Daim M.M., Gan S.H., Khalil M.I. (2017). Beneficial roles of honey polyphenols against some human degenerative diseases: A review. Pharm. Reports..

[14] Ferreres F., Tomás-Barberán F.A., García-Vignera C., Tomás-Lorente F. (1993). Flavonoids in honey of different geographical origin. Eur. Food Res. Technol..

[15] Pascual-Maté A., Osés S.M., Fernández-Muiño M.A., Sancho M.T. (2018). Methods of analysis of honey. J. Apic. Res..

[16] Pauliuc D., Dranca F., Oroian M. (2020). Antioxidant Activity, Total Phenolic Content, Individual Phenolics and Physicochemical Parameters Suitability for Romanian Honey Authentication. Foods.

[17] Escuredo O., Rodríguez-Flores M.S., Rojo-Martínez S., Seijo M.C. (2019). Contribution to the Chromatic Characterization of Unifloral Honeys from Galicia (NW Spain). Foods.

[18] Seijo M.C., Escuredo O., Rodríguez-Flores M.S. (2019). Physicochemical Properties and Pollen Profile of Oak Honeydew and Evergreen Oak Honeydew Honeys from Spain: A Comparative Study. Foods.

[19] Escuredo O., Rodríguez-Flores M.S., Meno L., Seijo M.C. (2021). Prediction of Physicochemical Properties in Honeys with Portable Near-Infrared (microNIR) Spectroscopy Combined with Multivariate Data Processing. Foods.

[20] Biesaga M., Pyrzyńska K. (2013). Stability of bioactive polyphenols from honey during different extraction methods. Food Chem..

[21] Cuevas-Glory L.F., Pino J.A., Santiago L.S., Sauri-Duch E. (2007). A review of volatile analytical methods for determining the botanical origin of honey. Food Chem..

[22] Vazquez L., Celeiro M., Sergazina M., Dagnac T., Llompart M. (2021). Optimization of a miniaturized solid-phase microextraction method followed by gas chromatography mass spectrometry for the determination of twenty four volatile and semivolatile compounds in honey from Galicia (NW Spain) and foreign countries. Sustain. Chem. Pharm..

[23] Singleton V.L., Rossi J.A. (1965). Colorimetry of total phenolics with phosphomolybdic-phosphotungstic acid reagents. Am. J. Enol. Vit..

[24] Brand-Williams W., Cuvelier M.E., Berset C. (1995). Use of a free radical method to evaluate antioxidant activity. LWT Food Sci. Technol..

[25] Celeiro M., Lamas J.P., Arcas R., Lores M. (2019). Antioxidants Profiling of By-Products from Eucalyptus Greenboards Manufacture. Antioxidants.

[26] Biesaga M., Pyrzynska K. (2009). Liquid chromatography/tandem mass spectrometry studies of the phenolic compounds in honey. J. Chromatogr. A.

[27] Pascual-Maté A., Osés S.M., Fernández-Muiño M.A., Sancho M.T. (2018). Analysis of Polyphenols in Honey: Extraction, Separation and Quantification Procedures. Sep. Purif. Rev..

[28] Attanzio A., Tesoriere L., Allegra M., Livrea M.A. (2016). Monofloral honeys by Sicilian black honeybee (*Apis mellifera* ssp. *sicula*) have high reducing power and antioxidant capacity. Heliyon.

