# Supplementary material for: Evaluating the Presence and Contents of Phytochemicals in Honey Samples: Phenolic Compounds as Indicators to Identify Their Botanical Origin
Source: Foods. 2021 Oct 28;10(11):2616. doi: 10.3390/foods10112616 (PMC8625021; doi:10.3390/foods10112616)
Supplement: Supplementary file 1 [file foods-10-02616-s001.zip › foods-1428582-supplementary.pdf]

Supplementary material

Evaluating the Presence and Contents of Phytochemicals in Honey Samples: Phenolic Compounds as Indicators to Identify Their Botanical Origin

Lua Vazquez [1], Daniel Armada [1], Maria Celeiro [1], Thierry Dagnac [2,*] and Maria Llompart [1,*]

[1] CRETUS, Department of Analytical Chemistry, Nutrition and Food Science, Universidade de Santiago de Compostela, E-15782 Santiago de Compostela, Spain; lua.vazquez.ferreiro@usc.es (L.V.); daniel.armada.alvarez@usc.es (D.A.); maria.celeiro.montero@usc.es (M.C.)

[2] Galician Agency for Food Quality-Agronomic and Agrarian Research Centre (AGACAL-CIAM), Unit of Food and Feed Safety and Organic Contaminants, Apartado 10, E-15080 A Coruña, Spain

* Correspondence: thierry.dagnac@xunta.gal (T.D.); maria.llompart@usc.es (M.L.)

Table S1. Concentration (μg g^{-1}) of phenolic compounds, TPC (mg GAE $100g^{-1}$) and AA (μmol TRE $100g^{-1}$) in honeys of the 2018 season.

**Compounds**	**EU1**	**BL1**	**CN1**	**CN2**	**CN3**	**CN4**	**CN5**	**HD1**	**HD2**	**HD3**	**HD4**	**HD5**	**HD6**	**HD7**	**HD8**	**HE1**	**HE2**
gallic acid		2.1	1.1			2.1		3.0	6.2	3.0	9.8	3.8	4.5	7.0	7.0		
phloroglucinic acid														1.4			
β-resorcylic acid/protocatechuic acid	0.35	3.2	2.5	0.41	0.55	2.9	0.81	4.9	7.6	3.3	6.6	5.9	7.8	6.9	9.4	0.43	0.55
protocatechualdehyde				0.21	0.17		0.19									0.22	0.23
gentisic acid	0.36	1.2	1.2	0.31	0.55	1.8	0.52	2.5	3.0	2.9	3.8	2.1	3.2	3.3	3.6	0.12	0.21
chlorogenic acid	0.051	0.061	0.050	0.07	0.047		0.05	0.092		0.10			0.07			0.11	0.050
3-hydroxybenzaldehyde																1.21	
4-hydroxybenzaldehyde				1.5												1.7	1.8
γ-resorcylic acid		0.07	0.10		0.12	0.11		0.11	0.050	0.12	0.067	0.070	0.089	0.11	0.061		
α-resorcylic acid		0.06	0.10		0.092	0.089		0.10		0.13	0.072	0.071	0.084				
veratricacid		1.9										1.8					
caffeic acid	0.10	0.57	0.58	0.16	0.41	0.33	0.27	0.40	0.28	0.57	0.18	0.48	0.11	0.32	0.079	0.12	0.17
p-coumaric acid	0.72	2.0	2.0	0.50	1.3	2.1	0.85	3.5	1.8	3.4	1.3	1.7	2.6	0.88	0.27	0.050	0.16
veratraldehyde	0.049											0.06				0.063	
4-anisaldehyde				0.41												0.59	0.77
quercetin	0.47	0.44	0.44		0.29	0.24		0.24	0.63	0.53	0.45	0.40	0.31	0.33	0.16		
kaempferol		0.47						0.30	0.23	0.31	0.22	0.38		0.25			0.25
apigenin	0.090	0.16	0.13	0.18	0.11	0.071	0.13	0.11	0.16	0.15	0.15	0.15	0.06	0.17	0.060	0.077	0.14
chrysin	1.6	4.9	2.9	2.4	3.3	2.4	3.0	4.2	3.2	4.4	1.8	4.2	1.5	3.8	0.99	1.9	1.8
trans-ferulic acid		0.62				0.36		0.64		0.77		0.68	0.32	0.66	0.27	0.16	0.15
p-hydroxybenzoic acid	1.0	2.0	2.4	3.2	1.3	2.4	1.3	1.8	1.6	2.9	1.7	1.9	1.6	1.5	0.85	3.7	4.0
3-hydroxyphenylacetic acid		29				8.0		10		3.7		0.66	11	1.9	2.0	242	125
vanillic acid		0.27				0.069		0.14	6.2	0.23		0.15	0.22	0.63	0.55	0.10	0.12
**∑ [Phenolic compounds]**	**4.9**	**49**	**14**	**9.4**	**8.2**	**23**	**7.1**	**32**	**25**	**27**	**26**	**24**	**33.0**	**29.3**	**35**	**252**	**135**
**TPC (mg GAE/100 g)**	**68**	**111**	**129**	**165**	**86**	**122**	**97**	**152**	**177**	**142**	**174**	**131**	**144**	**150**	**179**	**203**	**191**
**AA (μmol TRE/100g)**	**102**	**286**	**339**	**200**	**185**	**404**	**140**	**568**	**878**	**501**	**1006**	**548**	**615**	**830**	**907**	**224**	**234**

*EU: Eucalyptus; BL: Blackberry; CN: Chestnut; HD: Honeydew; HE: Heather.

Table S1. Continuation. Concentration (μg g^{-1}) of phenolic compounds, TPC (mg GAE $100g^{-1}$) and AA (μmol TRE $100g^{-1}$) in honeys of the 2018 season.

**Compounds**[1]	**MF1**	**MF2**	**MF3**	**MF4**	**MF5**	**MF6**	**MF7**	**MF8**	**MF9**	**MF10**	**MF11**	**MF12**	**MF13**	**MF14**	**MF15**	**MF16**	**MF17**	**MF18**
gallic acid	0.84		1.8	0.33	0.26		0.21			0.52	0.24	3.5	0.21	0.57	0.76		0.22	
phloroglucinic acid																		
β-resorcylic acid/protocatechuic acid	2.6	2.1	4.2	0.45	0.54	0.52	1.9	1.0	0.74	0.70	0.59	11	1.1	0.69	0.66	0.55	0.99	0.94
protocatechualdehyde	0.20	0.12		0.36	0.43	0.080	0.20	0.12	0.23	0.07	0.11		0.28	0.21	0.078	0.05	0.18	0.090
gentisic acid	1.2	2.0	0.92			0.27	5.2	2.0	0.15	1.3	0.16	1.7	2.9	0.54	0.22	0.15	2.6	2.2
chlorogenic acid	0.088	0.070		0.16	0.17	0.10	0.11	0.15		0.070		0.19	0.13	0.10	0.051	0.050	0.089	0.18
3-hydroxybenzaldehyde					0.97		0.12	0.17	0.48									
4-hydroxybenzaldehyde				0.20	1.4				2.0								0.10	0.13
γ-resorcylic acid	0.057	0.10					0.31	0.10		0.060			0.13				0.10	0.10
α-resorcylic acid	0.064	0.083	0.072				0.28	0.080		0.070			0.11				0.11	0.091
veratric acid																		
caffeic acid	0.38	0.19	0.47	0.43	0.64	0.11	0.31	0.049	0.051	0.10	0.39	0.24	0.34	0.35	0.18	0.19	0.22	0.15
p-coumaric acid	1.2	10	1.4	1.6	1.5	1.5	11	11	0.38	5.1	1.3	1.5	12	2.2	0.71	3.3	4.3	4.0
veratraldehyde	0.051		0.059	0.06						0.07			0.26	0.05	0.26	0.45	0.20	0.06
4-anisaldehyde	0.22			0.58		0.21			0.66				0.18	0.16				0.31
quercetin	0.42	0.39	0.38	0.53			0.65	0.35		0.44	0.60	0.47	0.67	0.76	0.59	0.33	0.34	0.29
kaempferol	0.36		0.33		0.71		0.33				0.36		0.31			0.30	0.22	
apigenin	0.11	0.056	0.14	0.17	0.43	0.081	0.10				0.23	0.17	0.12	0.13	0.17	0.09	0.14	0.09
chrysin	3.7	2.0	4.4	4.2	5.9	1.8	3.1	0.69	0.51	1.5	4.3	2.0	4.4	3.1	3.0	2.8	2.0	2.0
trans-ferulic acid		0.19	0.83									0.19	0.46			0.19		
p-hydroxybenzoic acid	3.5	2.1	2.8	3.3	3.8	1.9	3.2	3.0	4.6	1.9	1.0	2.6	3.6	2.1	1.3	4.1	5.1	4.2
3-hydroxyphenylacetic acid		21	8.3									8.3	6.9			20		
vanillic acid		0.11	0.069									0.40	0.52					
**∑ [Phenolic compounds]**	**15**	**40**	**26**	**12**	**17**	**6.5**	**27**	**19**	**9.8**	**12**	**9.3**	**33**	**35**	**11**	**8.0**	**33**	**17**	**15**
**TPC (mg GAE/100 g)**	**156**	**133**	**102**	**142**	**136**	**109**	**123**	**145**	**176**	**116**	**79**	**136**	**142**	**115**	**83**	**97**	**145**	**148**
**AA (μmol TRE/100g)**	**324**	**283**	**311**	**139**	**175**	**127**	**282**	**249**	**241**	**180**	**121**	**484**	**249**	**104**	**127**	**105**	**185**	**183**

*MF: Multi-floral.

Table S2. Concentration (μg g^{-1}) of phenolic compounds, TPC (mg GAE $100g^{-1}$) and AA (μmol TRE $100g^{-1}$) in honeys of the 2019 season.

**Compounds**	**EU2**	**EU3**	**EU4**	**EU5**	**EU6**	**EU7**	**EU8**	**EU9**	**EU10**	**EU11**	**EU12**	**BL2**	**BL3**	**BL4**	**BL5**	**BL6**	**BL7**	**BL8**
gallic acid			0.19	0.27	0.22	0.34			0.47	0.20	0.13	0.33	0.46	1.6	0.29	0.37	2.0	0.66
phloroglucinic acid													0.21	0.34				
β-resorcylic acid/protocatechuic											0.22	0.50	0.88	2.9	0.96	0.42	4.6	2.0
acid	0.32	0.54	0.49	0.48	0.55	0.32	0.46	0.49	0.36	0.26								
caftaric acid	0.061	0.10										0.05						
protocatechualdehyde	0.075	0.14	0.27	0.18	0.080	0.091	0.090	0.048				0.15	0.15		0.19	0.13		0.069
gentisic acid	0.11	0.081	0.12	0.10	0.44	0.23	0.10	0.16		0.071	0.10	0.17	0.43	1.1	0.30	0.10	1.3	0.97
gentisaldehyde	0.31	0.49	0.54															
chlorogenic acid		0.10	0.078			0.10	0.10						0.05	0.071	0.062	0.090		0.070
3-hydroxybenzaldehyde		0.85	0.51	0.87		0.13							0.22		0.10			
4-hydroxybenzaldehyde		1.3	1.3	1.2			1.3						0.28					
γ-resorcylic acid	0.051				0.060			0.073				0.050		0.089		0.05	0.081	0.069
α-resorcylic acid					0.062			0.067					0.051	0.080		0.05	0.070	0.062
veratric acid																1.2		
caffeic acid	0.17	0.11	0.37	0.37	0.32	0.25	0.12	0.23	0.068	0.080	0.17	0.81	0.47	0.57	0.58	0.44	0.35	0.59
p-coumaric acid	0.78	0.48	1.4	1.2	2.4	4.2	0.92	0.76	0.52	0.59	1.6	0.44	2.6	2.1	3.8	1.7	0.98	4.1
veratraldehyde	0.083	0.080	0.067	0.060	0.12	0.05							0.09					
4-anisaldehyde			0.28															
quercetin	0.51			0.25	0.66	0.80			0.28		0.59		1.2	0.48	1.0	1.1	0.25	1.4
kaempferol	0.25			0.44	0.23								0.46	0.25	0.49	0.32		0.43
apigenin	0.10	0.075	0.16	0.39	0.11	0.25	0.14	0.13			0.06	0.17	0.26	0.23	0.23	0.31	0.13	0.26
chrysin	1.9	0.89	2.9	3.4	2.1	2.9	1.2	2.0	0.50	0.87	1.9	2.2	5.5	3.2	3.5	2.7	2.5	5.9
trans-ferulic acid	0.71	0.54	0.88	0.88	0.91	0.87	0.53	0.69	0.51	0.48	0.61	0.67	1.5	1.0	1.7	1.3	0.90	1.3
p-hydroxybenzoic acid	0.77	1.7	1.7	2.3	1.3	1.5	2.3	0.92	1.2	0.85	0.97	1.5	2.3	1.2	0.88	0.78	0.68	1.8
3-hydroxyphenylacetic acid		61	53	80			123		12			64						3.7
vanillic acid																		
**∑ [Phenolic compounds]**	**6.2**	**68**	**64**	**93**	**9.6**	**12**	**130**	**5.5**	**16**	**3.4**	**6.4**	**71**	**17**	**15**	**14**	**11**	**13.9**	**2**
**TPC (mg GAE /100 g)**	**61**	**139**	**127**	**115**	**64**	**105**	**124**	**61**	**58**	**48**	**52**	**48**	**97**	**95**	**57**	**61**	**111**	**98**
**AA (μmol TRE/100g)**	**151**	**249**	**846**	**143**	**104**	**201**	**363**	**353**	**312**	**15**	**28**	**201**	**220**	**164**	**111**	**186**	**428**	**260**

*EU: Eucalyptus; BL: Blackberry.

Table S2. **Continuation.** Concentration (μg g^{-1}) of phenolic compounds, TPC (mg GAE $100g^{-1}$) and AA (μmol TRE $100g^{-1}$) in honeys of the 2019 season.

Compounds	CN6	CN7	CN8	CN9	CN10	CN11	CN12	CN/ HD1	CN/ HD2	CN/ HD3	CN/ HD4	CN/ HD5	CN/ HD6	CN/ HD7	HD9	HD10	HD11	HD12	HE3	HE4
gallic acid	0.46	0.48	0.18	0.29	0.32	3.3	0.18	1.6	6.8	1.7	5.4	3.8	5.7	5.2	1.6	7.2	6.7	6.6	1.1	0.20
phloroglucinic acid	0.13		0.11					0.29	0.42	0.29	0.87				0.31				0.38	
β-resorcylic acid/protocatechuic acid	1.4	1.3	0.37	0.40	0.64	5.9	0.67	2.5	8.0	2.7	7.2	4.8	7.2	6.1	6.2	10	9.6	5.7	2.5	0.36
cafftaric acid																				
protocatechualdehyde	0.090	0.10	0.22	0.25	0.24		0.093	0.054												0.16
gentisic acid	1.2	1.1	0.16	0.31	0.37	2.0	1.3	1.8	2.6	1.7	3.0	2.6	2.9	2.5	1.6	2.3	2.2	2.0	0.98	0.14
gentisaldehyde	0.21		0.43	0.24	0.18							0.64							0.32	
chlorogenic acid	0.071	0.056	0.10	0.069	0.054		0.18	0.15		0.064								0.06		0.12
3-hydroxybenzaldehyde							0.30									0.10				0.73
4-hydroxybenzaldehyde		0.33	0.67				0.35													1.2
γ-resorcylic acid	0.082	0.10	0.050	0.10	0.11	0.073	0.066	0.10		0.10	0.10	0.10	0.083	0.058	0.12	0.070		0.05	0.08	
α-resorcylic acid	0.078	0.11		0.10	0.10	0.077	0.092	0.10		0.10	0.10	0.081	0.055	0.053	0.11	0.064			0.07	
veratricacid					1.6															
caffeic acid	0.44	0.27	0.21	0.69	0.85	0.26	0.06	0.33	0.28	0.57	0.24	0.62	0.35	0.22	0.43	0.13	0.073	0.31	0.42	0.17
p-coumaric acid	5.1	4.5	5.4	3.2	1.4	1.8	11	6.7	1.4	5.5	1.6	4.5	1.6	1.7	1.4	1.6	0.59	1.4	2.6	0.65
veratraldehyde															0.070					0.10
4-anisaldehyde		0.13					0.24													0.14
quercetin	0.59	0.45	1.3	0.28	0.33	0.43	0.22	0.42	0.39	0.62		0.46	0.39		0.31		0.22	0.29	0.79	
kaempferol			0.60		0.44	0.27		0.32		0.41		0.37		0.24				0.26	0.31	
apigenin	0.17	0.083	0.31	0.23	0.20	0.090	0.10	0.16	0.10	0.21	0.10	0.20	0.10	0.091	0.17	0.059		0.20	0.19	0.11
chrysin	3.5	1.7	4.5	3.4	3.6	2.1	0.53	3.3	2.1	4.2	2.1	2.9	1.7	2.2	2.5	1.2	0.94	2.4	3.1	2.4
trans-ferulic acid	0.96	0.83	2.0	1.1	1.2	0.58	0.44	0.73	0.78	0.92	0.74	1.0	0.42	0.43	0.96	0.62	0.31	0.86	1.1	0.51
p-hydroxybenzoic acid	1.4	3.4	1.2	1.3	1.7	2.5	1.8	2.4	1.4	2.5	1.7	2.2	1.1	1.7	2.1	1.6	0.83	0.98	1.4	1.6
3-hydroxyphenylacetic acid				2.8		12							1.7	9.5			0.41			54
vanillic acid						0.20							0.15	0.40			0.37			
**∑ [Phenolic compounds]**	**16**	**15**	**18**	**14.7**	**13**	**31**	**18**	**21**	**26**	**22**	**23**	**26**	**23**	**30**	**18**	**25**	**22**	**21**	**15**	**62**
**TPC (mg GAE/100 g)**	**134**	**112**	**117**	**69.4**	**64**	**129**	**192**	**135**	**160**	**119**	**147**	**125**	**192**	**159**	**95**	**172**	**110**	**197**	**102**	**156**
**AA (μmol TRE/100g)**	**315**	**277**	**220**	**194**	**199**	**540**	**163**	**485**	**697**	**228**	**611**	**427**	**845**	**854**	**420**	**874**	**1017**	**903**	**392**	**138**

CN: Chestnut; CN/HD: Chestnut/Honeydew; HD: Honeydew; HE: Heather.

Table S2. **Continuation.** Concentration (μg g^{-1}) of phenolic compounds, TPC (mg GAE $100g^{-1}$) and AA (μmol TRE $100g^{-1}$) in honeys of the 2019 season.

**Compounds**	**MF19**	**MF20**	**MF21**	**MF22**	**MF23**	**MF24**	**MF25**	**MF26**	**MF27**	**MF28**	**MF29**	**MF30**	**MF31**	**MF32**	**MF33**	**MF34**	**MF35**	**MF36**
gallic acid		0.20	0.46	0.24	1.1	0.19	0.35	0.27	0.54	0.15	0.22	0.15	0.53	4.5	0.16	1.7	0.24	
phloroglucinic acid	0.18	0.32	0.32															
β-resorcylic acid/protocatechuic acid	0.94	0.79	1.9	0.35	2.9	0.54	0.82	0.50	0.39	0.52	0.26	0.38	0.78	6.4	0.52	4.7	0.33	0.42
cafftaric acid			0.078															
protocatechualdehyde	0.13	0.19	0.11	0.052		0.12	0.13	0.18	0.11		0.16	0.14	0.070				0.080	0.11
gentisic acid	0.59	0.97	3.5		0.81	0.22	1.9	0.42	0.61		0.16	0.063	0.98	2.7	0.11	2.1	0.41	0.20
gentisaldehyde		0.22								0.27								
chlorogenic acid	0.07	0.054	0.070	0.071		0.066	0.08	0.067	0.10					0.062			0.048	
3-hydroxybenzaldehyde																		
4-hydroxybenzaldehyde	0.32					1.5	0.23			1.5								
γ-resorcylic acid	0.051	0.057	0.18		0.10		0.13	0.052			0.063		0.12	0.078		0.068		
α-resorcylic acid	0.060	0.072	0.20		0.090		0.11	0.063	0.078		0.088		0.13	0.061	0.06	0.067	0.051	0.063
veratricacid								0.89										1.3
caffeic acid	0.43	0.23	0.40	0.16	0.55	0.079	0.15	0.44	0.28	0.053	0.35	0.26	0.25	0.43	0.18	0.27	0.20	0.31
p-coumaric acid	3.4	8.2	4.7	1.5	2.3	0.63	10	3.5	2.0	0.14	1.2	0.95	1.7	10	6.2	7.6	4.4	1.3
veratraldehyde	0.055															0.050		
4-anisaldehyde	0.12								0.13	0.72								
quercetin	0.48	0.58	0.81		0.83		0.91	1.1	0.47		0.45	0.32		0.61	1.0		0.63	0.38
kaempferol	0.24		0.26		0.32			0.34				0.23		0.39	0.46			
apigenin	0.12	0.092	0.14	0.13	0.23	0.073		0.32	0.18	0.045	0.09	0.17	0.08	0.17	0.26	0.10	0.10	0.15
chrysin	3.2	1.5	3.4	1.6	3.7	0.72	1.7	4.1	2.2	0.70	2.4	2.4	2.8	4.2	2.0	1.8	2.3	2.8
trans-ferulic acid	1.5	0.64	0.32	0.69	1.2	0.47	0.60	0.97	0.65	0.36	0.77	0.75	0.68	0.58	0.57	0.34	0.81	0.92
p-hydroxybenzoic acid	2.5	1.5	2.0	1.4	1.8	2.5	2.0	1.8	1.4	2.3	0.86	0.77	0.96	2.3	1.5	1.5	1.5	0.71
3-hydroxyphenylacetic acid			1.3	17						138				5.0		12		
vanillic acid														0.58		0.15		
**∑ [Phenolic compounds]**	**14**	**16**	**20**	**23**	**16**	**7.2**	**19**	**15**	**9.2**	**144**	**7.1**	**6.6**	**9.1**	**38**	**13**	**32**	**11**	**8.6**
**TPC (mg GAE/100 g)**	**117**	**83**	**98**	**78**	**91**	**170**	**193**	**90**	**111**	**144**	**61**	**55**	**113**	**13**	**56**	**133**	**87**	**67**
**AA (μmol TRE/100g)**	**291**	**263**	**837**	**104**	**231**	**256**	**287**	**387**	**371**	**168**	**59**	**56**	**138**	**562**	**178**	**490**	**196**	**313**

*MF: Multi-floral.
